# Déterminants de l’acceptation de la vaccination à Makokou au Gabon

**DOI:** 10.11604/pamj.2024.49.71.41612

**Published:** 2024-11-08

**Authors:** Ulrick Jolhy Bisvigou, Edgard Brice Ngoungou, Steeve Minto'o Rogombe, Sydney Maghendji Nzondo, Euloge Ibinga

**Affiliations:** 1 Département d'Épidémiologie, Biostatistiques et Informatique Médicale, Santé Publique, Médecine du Travail et Médecine Légale, Faculté de Médecine, Université des Sciences de la Santé, Libreville, Gabon,; 2 Unité de Recherche en Épidémiologie des Maladies Chroniques et Santé Environnement, Faculté de Médecine, Université des Sciences de la Santé, Libreville, Gabon,; 3 Département de Pédiatrie, Faculté de Médecine, Université des Sciences de la Santé, Libreville-Owendo, Gabon

**Keywords:** Gabon, programme élargi de vaccination, enquête, hésitation vaccinale, Gabon, expanded program on immunization, survey, vaccination hesitancy

## Abstract

**Introduction:**

les motifs d'hésitation vaccinale dans la communauté doivent être identifiés afin d'y remédier. L'analyse des connaissances, attitudes et perceptions des parents, permettrait d'expliquer les faibles performances du Programme élargi de vaccination (PEV).

**Méthodes:**

une enquête auprès des ménages de la ville de Makokou a été réalisée durant le mois d'octobre 2021. Le questionnaire portait sur cinq domaines de la vaccination: accès, accessibilité, information, acceptation, activation. Une analyse descriptive puis une régression logistique ont été réalisées pour mesurer l'effet de l'acceptation.

**Résultats:**

au total 486 personnes en milieux urbain (66%) et rural (34%), ont été interrogées. Les participants étaient âgés de 18 à 86 ans; l'âge moyen était de 28,5±11,3 ans, le sexe-ratio de 0,69. Le nombre d'enfants à charge variait de 0 à 21, avec une moyenne de 1,3 enfants. La majorité des personnes interrogées (61,9%) ne connaissent pas le PEV; la maladie la plus reconnues étaient le tétanos (37%), la principale source d'information était la discussion familiale (40,5%). Les situations gênantes au centre de vaccination étaient le temps d'attente (51,9%), le manque d'information (26,7%) et le mauvais accueil (20,2%). Près de la moitié (48%) ne faisaient pas confiance aux vaccins du gouvernement. Le sexe masculin, le milieu de vie rural, la difficulté d'accès, le manque d'information étaient les principaux facteurs associés à l'acceptation de la vaccination dans l'Ogooué-Ivindo.

## Abstract (en)

**Conclusion:**

de nombreuses connaissances erronées dans les communautés de Makoukou nécessitent d'être corrigées par le PEV, afin d'intégrer une culture de vaccination.

**Introduction:**

the root causes of vaccine hesitancy in communities must be determined in order to address them. Analyzing the knowledge, attitudes, and perceptions of parents could help explain the poor performance of the Expanded Program on Immunization (EPI).

**Methods:**

a household survey was conducted in the city of Makokou in October 2021. The questionnaire covered five areas of vaccination: access, accessibility, information, acceptance and activation. A descriptive analysis followed by logistic regression was performed to measure the effect of vaccine acceptance.

**Results:**

a total of 486 people, from urban (66%) and rural (34%) areas, were surveyed. Participants ranged in age from 18 to 86 years old, with an average age of 28.5±11.3 years, and a sex ratio of 0.69. The number of dependent children ranged from 0 to 21, with an average of 1.3 children. The majority of respondents (61.9%) were unaware of the EPI; the most recognized disease was tetanus (37%) and the main source of information was family discussion (40.5%). The main issues at vaccination centers were waiting times (51.9%), lack of information (26.7%) and poor reception (20.2%). Nearly half (48%) did not trust government vaccines. Male gender, rural living environment, difficulty in accessing the centers, and lack of information were the main factors associated with vaccine acceptance in Ogooué-Ivindo.

**Conclusion:**

numerous misconceptions in the communities of Makokou need to be corrected by the EPI to foster a culture of vaccination.

## Introduction

Le Programme élargi de vaccination (PEV) a contribué de manière significative aux réductions de la morbidité et de la mortalité infantiles dans le monde et surtout dans les pays en voie de développement [1,2]. Ce programme avait été créé en 1974, lors de la conférence d'Alma-Ata, pour développer et étendre des programmes de vaccination à travers le monde, après le succès majeur de la variolisation [3]. En 1974, moins de 5% des enfants des pays en voie de développement recevaient la 3^e^ dose de vaccins DTCP (Diphtérie, Tétanos, coqueluche et antipoliomyélitique) au cours de leur 1^ère^ année de vie [4]. Ces faibles couvertures vaccinales étaient dues à de nombreuses raisons, notamment financières. En 1977, l'objectif fixé par le PEV était de rendre la vaccination contre la diphtérie, la coqueluche, le tétanos, la poliomyélite, la rougeole et la tuberculose, accessible à tous les enfants dans le monde à l'horizon 1990. Cependant, la proportion d'enfants qui terminent le calendrier de vaccination recommandé n'a pas augmenté dans le monde comme prévu.

Le Gabon n'est pas un pays éligible à *GAVI* (« Global Alliance for Vaccines and Immunization », en français, l'Alliance globale pour les vaccins et l'immunisation) car il s'agit d'un pays à revenu intermédiaire de la tranche supérieure, selon la Banque mondiale [5]. Le Gabon est l'un des pays de la région africaine qui finance totalement et sur fonds propres son programme de vaccination, notamment l'achat des vaccins et consommables de la vaccination, avec l'aide de partenaires tels que l'OMS et l'Unicef. Dans les centres publics dédiés, la vaccination est gratuite pour la cible, c'est-à-dire les enfants de moins d'un an, les femmes en âge de procréer et les femmes enceintes. Le programme de vaccination de routine au Gabon recommande et prend en charge différents vaccins pour la prévention des maladies évitables par la vaccination: le Bacille de Calmette-Guérin (BCG), contre la tuberculose, les vaccins Polio Oral (VPO) et polio injectable (VPI), contre la poliomyélite, le vaccin Pentavalent contenant cinq antigènes (contre la diphtérie, le tétanos, la coqueluche, l'hépatite B, et *Haemophilus influenzae* type b), le vaccin contre la rougeole (VAR), le vaccin contre la fièvre jaune (VAA). Les vaccins précités sont administrés aux enfants de moins d'un an selon un calendrier vaccinal prévoyant 10 rendez-vous: à la naissance, 6 semaines, 10 semaines, 4 semaines, 9 mois et 12 mois. Les femmes enceintes et en âge de procréer ne reçoivent que le vaccin antitétanique. Leur calendrier vaccinal prévoit 5 rendez-vous: dès que possible pour les femmes enceintes et celles en âge de procréer, 1 mois après la première dose, 6 mois après la deuxième dose, 1 an après la troisième dose, 1 an après la quatrième dose. Le programme assure la disponibilité des vaccins dans tous les centres de vaccination publiques de toutes les 10 régions sanitaires du Gabon. A côté de cela, les données administratives montrent que la couverture vaccinale reste en deçà de la norme fixée par l'OMS qui est de 80% dans les districts ou départements et de 90% au niveau national. Les objectifs fixés de couverture vaccinale n'ont toujours pas été atteints. Une revue du PEV, réalisée en 2012, l'EDS-II 2012 (Enquête démographie et Santé 2012), montrent que ces objectifs non atteints, seraient liées à l'accès, au coût indirect des vaccins, à la communication délivrée aux populations ou à d'autres facteurs culturels ou comportementaux, qui interviendraient dans la décision de se faire vacciner ou de faire vacciner son enfant [6,7].

Une étude a été mené pour identifier les facteurs qui pourraient, dans la perception de l'acte vaccinal en population générale, dans la région Est du Gabon, expliquerai le faible taux de couverture vaccinale des enfants de moins de 1 an et des femmes enceintes, cibles du Programme élargi de vaccination, observé depuis plusieurs années.

## Méthodes

**Lieu de l'étude**: cette étude a été menée dans la province de l'Ogooué-Ivindo qui compte environ 73.979 habitants, répartis sur quatre départements (Ivindo, Mvoung, Lopé et Zadié). Le PEV est disponible dans cinq structures sanitaires que sont les trois centres médicaux (Mékambo, Ovan et Booué), le Centre de santé urbain de Makokou et l'Hôpital Régional de Makokou. La couverture vaccinale du vaccin pentavalent (vaccin contenant les antigènes contre la diphtérie, le tétanos, la coqueluche, l'hépatite B et les infections à *Haemophilus influenzae* type b), notamment la 3^e^ dose, est très faible, 52% dans l'Ivindo, 23% dans la Zadié, par exemple, selon les données du ministère de la Santé en mai 2021.

**Type et période de d'étude**: il s'agit d'une enquête transversale descriptive, à visée analytique, en population générale, durant le mois d'octobre 2021. Il s'agit d'une enquête de type CAP (Connaissance Attitude et Pratiques), réalisée à l'aide d'un questionnaire, au cours d'un entretien face à face.

**Participants**: les participants à cette étude sont des personnes vivant dans l'Ogooué-Ivindo, dans la ville de Makokou et ses environs, en zone rurale et urbaine, avec un âge supérieur ou égal à 18 ans, quel que soit le sexe, avec ou sans enfants.

**Technique d'échantillonnage et taille de l'échantillon**: le nombre de sujet nécessaire est calculé à partir de la formule de Lorentz. En considérant p la proportion de personnes qui acceptent la vaccination à 50%, t=1,96 et l'erreur standard e=0,05, le nombre de sujet nécessaire est de 384,16.

**Collecte de données**: l'équipe de terrain est constituée de 5 enquêteurs, formés durant une journée entière, à l'utilisation de l'outil de collecte, installé sur un smartphone, et à la méthodologie de cette enquête. La formation porte également sur la stratégie de l'entretien, pour obtenir des réponses sincères des enquêtés lors des entretiens face à face, et sur le respect des considérations éthiques.

Les données sont recueillies à l'aide d'une application mobile connecté à une base de données (formulaire Google form®) installée sur leurs téléphones portables. Les enquêteurs recevaient chaque jour du crédit téléphonique et internet pour pouvoir se connecter et implémenter la base de données en ligne. Dans ce système d'information connecté, pour garantir l'anonymat, les participants n'étaient identifiés qu'à travers les initiales du nom et du prénom, tenant sur deux lettres. Les données à caractère personnelles étaient recueillies dans un registre, ainsi que le consentement éclairé signé sur le terrain. La qualité des données a été assurée par la supervision sur place et l'examen des formulaires remplis quotidiennement.

Les réponses aux questions recueillies sur la plateforme mobile, portaient sur les données sociodémographiques (l'âge, le sexe, le lieu de résidence, la religion, le statut matrimonial, le nombre d'enfants en charge, la profession, le niveau d'études) et cinq (5) domaines, appelées les « 5A » ou « Five A's » en anglais.

**Outil de mesure *Five A*:** l'objectif de la méthode *Five A* de mesurer les attitudes, les perceptions face à la vaccination afin d'identifier les barrières ou les leviers rationnels ou émotionnels, guider les changements de comportement et prédire les résultats des interventions.

Ces 5 domaines sont, le niveau d'information sur la maladie et sa prise en charge (connaissance du nom, des symptômes, du vaccin, de la campagne de vaccination, des vaccins du PEV et de la source d'information); l'accès (l'accès facilité à un centre de vaccination) ; l'accessibilité (coût indirect de l'achèvement de la vaccination), activation (si les personnes interviewées sont prêtes à promouvoir la vaccination autour d'elles), l'acceptation (la vaccination est acceptée par les populations). L'initiative des “5A” est une démarche stratégique d'analyse et de planification structurée visant à identifier les causes fondamentales d'une faible couverture vaccinale [7-9]. Pour évaluer le niveau d'acceptation, le participant avaient donné une note comprise entre 1 et 10. Le questionnaire avait été au préalable validé par les concepteurs de l'initiative. Le questionnaire utilisé lors de l'enquête est un questionnaire qui avait été validé.

**Analyse de données**: les données enregistrées sur la plateforme ont été importées au format .csv. L'analyse a été réalisée à l'aide des logiciels R version 3.6.3 et RSutio version 1.4.1717.

Nous avons effectué une analyse descriptive, les variables qualitatives ont été décrite en effectifs et pourcentage, les variables quantitatives en moyenne et écart-type. Le test de Student était utilisé pour comparer les variables quantitatives, le test du chi-2 pour les variables qualitatives. Une association a été recherchée entre l'acceptation de la vaccination et les autres variables.

Une analyse de régression logistique, en utilisant la méthode pas à pas descendante, a été réalisée pour mesurer l'effet de chacun des déterminants identifiés sur l'acceptation de la vaccination. La variable à expliquer était l'acceptation de la vaccination. Les autres variables ont été introduites dans le modèle initial, par rapport à leur association avec l'acceptation de la vaccination, puis retirées du modèle en suivant la méthode pas à pas descendante. Le modèle de régression final a été obtenu en recherchant le résidu le plus faible possible. Le seuil de significativité statistique a été fixé à alpha= 0,05.

**Considérations éthiques**: l'équipe qui a collecté les données a adhéré aux normes éthiques internationales et à la Déclaration d'Helsinki. Une formation complémentaire a été réalisée afin que ces normes soient respectées tout au long de l'étude. Les participants ont été informés que la participation était volontaire, qu'ils pouvaient se retirer à tout moment et que les réponses seraient confidentielles. Les participants ont également reçu les coordonnées du personnel de l'Université des Sciences de la Santé d'Owendo qui pilotait cette enquête. Ils avaient la possibilité d'entrer en contact avec eux et poser des questions sur l'éthique ou le contenu de la recherche. Un consentement éclairé était demandé et signe avant l'administration du questionnaire. L'ensemble de données que nous avons utilisé pour l'analyse statistique ne contenait pas d'informations à caractère personnel ou pouvant permettre d'identifier formellement les participants. Les noms et numéros de téléphone des participants ne figuraient que dans un registre utilisé par les enquêteurs. Ces informations ne figuraient que dans le formulaire et le registre de consentement éclairé, signés par le participant.

## Résultats

**Description des participants:** au total 486 personnes ont été interviewées, provenant des zones urbaines (320 personnes, 66%) et rurales (166 personnes, 34%), à Makokou et ses alentours. Les participants étaient âgés de 18 à 86 ans. L'âge moyen était de 28,5 ± 11,3 ans. Le sexe-ratio était de 0,69 (Tableau 1).

**Tableau 1:** caractéristiques sociodémographiques des participants à l'enquête en octobre 2021 (n=486)

	Effectifs	%
**Milieu**		
Rural	166	34,2
Urbain	320	65,8
**Classe d’age**		
<25	228	46,9
25-35	163	33,5
35-45	43	8,8
45-55	33	6,8
55-et plus 65	19	4,0
**Sexe**		
Masculin	199	41,0
Féminin	287	59,0
**Statut matrimonial**		
Célibataire	382	78,6
Divorcé(e)	3	0,6
En couple (marié et concubinage)	99	20,4
Veuf (veuve)	2	0,4
**Religion**		
Bwitiste-animiste	33	6,8
Chrétien	402	82,7
Musulman	22	4,5
Autres	29	6,0
**Statut dans le foyer**		
Autres	65	13,4
Enfant	188	38,7
Epouse	38	7,8
Mère célibataire	123	25,3
Père, chef de foyer	72	14,8
**Niveau scolaire**		
Non scolarisé	41	8,4
Primaire	46	9,5
Secondaire	287	59,1
Supérieur	112	23,3

**Connaissance sur le PEV et les maladies évitables par la vaccination:** des personnes interrogées, 301 (61,9%) ne savent pas ce qu'est le Programme Élargi de Vaccination (PEV). Des 10 maladies évitables par la vaccination, ciblées par le PEV du Gabon, les quatre les plus connues étaient le tétanos (182, 37%), la tuberculose (156, 32%), la rougeole (156, 32%) et la fièvre jaune (141, 29%). La poliomyélite, qui fait pourtant l'objet d'un programme d'éradication, venait en 6 *^e^* position (91, 19%) ; la moins connues étaient la diphtérie (45, 9,3%). Il faut noter que 20% des interviewés pensaient que ces maladies avaient disparu (Tableau 2).

**Tableau 2:** niveau de connaissance sur les maladies évitables par la vaccination (MEV), ciblées ou non par le PEV des personnes interrogées à Makokou dans la population de l'Ogooué-Ivindo en octobre 2021

Question	Effectifs	%
**Quelle est votre source d’information sur la vaccination?**		
Discussion familiale	197	43.2
Média traditionnels (radio, télévision, journaux)	125	27.4
Personnel de santé	95	20.8
Internet	33	7.2
Lecture du carnet de santé	24	5.3
Leader politique ou religieux	45	9.9
Autre	9	2.0
**Est-il important d’être vaccine contre toutes ces maladies?**		
Oui	197	40.5
Non	73	15.0
Ne sait pas	137	28.2
Non, seulement pour certains	79	16.4
**Quelle idée aviez-vous de la vaccination en général, avant le début de la pandémie?**		
Réservée aux personnes d’un certain âge	282	58.1
Pour tout le monde quel que soit l’âge	74	15.3
Réservée aux voyageurs	56	11.5
Réservée aux personnes malades	29	6.0
Réservée à certaines professions	43	8.9
Aucune idée	42	8.7
Autres	6	1.2
**Connaissez-vous le Programme Elargi de Vaccination (PEV) ?**		
Non	301	61.9
Oui	52	10.7
Vaguement	133	27.4
**Savez-vous où se fait la vaccination du PEV**		
Oui	83	17.0
Non	272	56.0
Vaguement	131	27.0
**Etes-vous au courant des campagnes de vaccination organisées par le Ministère de la santé?**		
Oui, toujours	68	14.0
Oui, parfois	277	57.0
Non, jamais	98	20.2
Ne sait pas	43	8.8
**Avez-vous déjà entendu des rumeurs au sujet des vaccins du PEV ?**		
Oui	65	13.4
Non	239	49.3
Pas encore	182	37.3
**Avez-vous entendu des rumeurs au sujet d’autres vaccins?**		
Oui	283	58.2
Non	33	6.8
Pas encore	170	35.0
**Que pensez-vous au sujet de ces rumeurs?**		
Elles sont vraies	78	16.0
Elles sont fondées et possibles	76	15.6
Elles sont fausses	116	23.9
Ne sait pas	216	44.5
**Jusqu'à quel âge pensez-vous qu’on doit se faire vacciner ?**		
1 an	19	3.9
Adolescence	95	19.5
Age adulte	170	35.0
Seulement à la naissance	108	22.2
Ne sait pas	2	0.4
Pas besoin	92	18.9
**Savez-vous qu'il existe d’autres vaccins en dehors de ceux du PEV ?**		
Non	393	80.9
Oui	93	19.1
**Si Oui lesquels ?**		
Contre la rage	2	4.0
Contre le paludisme	2	4.0
Contre le coronavirus et la poliomyélite	2	4.0
Contre le paludisme et le tétanos	1	2.0
Contre la poliomyélite	5	10.0
Contre la rougeole	1	2.0
Contre le tétanos et la grippe	1	2.0
Contre le coronavirus	24	49.0
Contre le cancer de l'utérus	2	4.0
Ne sait pas	9	18.0

Les principales sources d'information sur la vaccination étaient les discussions familiales. La majorité (40,5%) pensait qu'il est important d'être vacciné et que la vaccination était réservée aux personnes d'un certain âge (58,1%), les enfants de moins d'un an. Il existerait des rumeurs au sujet du PEV (13,4%) et au sujet des vaccins (58,2%). Ces rumeurs seraient vraies selon 16% de la population interrogée. Très peu d'entre eux savait qu'il existe d'autres vaccins en dehors du PEV (19,1%) ; le plus connu des vaccins hors PEV était le vaccin contre le coronavirus, ceci était probablement dû à l'actualité. L'utilité des vaccins, d'après les populations, était de permettre d'être en bonne santé (35,1%).

**Accès aux services de vaccination**: la fréquentation des centres de vaccination n'était pas régulière, elle était occasionnelle dans 40,3% des cas. Cette fréquentation n'était pas influencée par une personne extérieure, elle se faisait surtout par habitude (37,2%). Les obstacles à cette fréquentation étaient l'éloignement des centres de vaccination (25,5%), le coût des vaccins ou de la vaccination (24,2%) et le mauvais accueil du personnel de santé (23,8%). La plupart des personnes interrogées n'avaient pas fait vacciner leur enfant à la dernière campagne (71,6%), (Tableau 3). Les situations gênantes qui pourraient être à l'origine de l'abandon de la vaccination étaient le temps d'attente long (51,9%), le manque d'information (26,7%) et le mauvais accueil (20,2%).

**Tableau 3:** perceptions de l'accès aux services de vaccination en octobre 2021 à Makokou

Question	Effectifs	%
**A quelle fréquence faites-vous vacciner vos enfants?**		
Régulièrement	135	27,7
Occasionnellement	196	40,3
Jamais	30	6,2
Ne sait pas	98	20,2
Pas d’enfants	27	5,6
**Qu’est-ce qui vous a amené, influencé, dans le choix de faire vacciner vos enfants?**		
Vous-même par habitude	181	37,2
Votre conjoint	39	8,0
La publicité à la télévision	69	14,2
Le personnel de santé	71	14,6
Mon entourage (famille, amis, collègue)	123	25,3
Autre	3	0,6
**Quelles sont les raisons pour lesquelles votre enfant n’est pas régulièrement vacciné?**		
Méfiance envers les vaccins	81	16,7
Éloignement des centres de vaccination	124	25,5
Coût des vaccins	117	24,2
Mauvais accueil	115	23,8
Méfiance envers les vaccins	81	16,7
Négligence	53	11,0
Coût du transport	39	8,1
Autre	5	1,0
**Avez-vous fait vacciner votre enfant lors de la dernière campagne gouvernementale contre la poliomyélite?**		
Oui	94	19,3
Non	348	80,7
**Si non pourquoi?**		
Pas d’enfant	57	11,7
J'étais en déplacement	23	4,7
Je n'ai pas été informé	1	0,2
La peur	4	0,8
Manque de confiance	1	0,2
Parce qu’il n’y a pas le temps	1	0,2
A cause des rumeurs	3	0,6
Il n'était pas encore né	310	63,8
Pas de raison	2	0,4
Raisons personnelles	3	0,6
**Si Oui, Pourquoi?**		
A cause du faible coût de transport	6	1,2
Aucune idée	417	85,8
Bon accueil bonne humeur générosité	18	3,7
Car c’est le meilleur de la région	8	1,6
Le centre de référence ou unique centre	5	1,0
Les sont vaccinés lors du porte à porte	1	0,2
Par ce que c’est le plus proche	11	2,3
Parce que c'est le plus propre et ordonné	3	0,6
Parce que ce le meilleur endroit où je peux vacciner mes enfants	1	0,2
Parce qu’il est	1	0,2
Parce que toute la famille se fait vacciner là-bas	1	0,2
Question de fiabilité prudente bonne conseil soins appropriés	7	1,4
Raisons personnelles	2	0,4
Rien à dire	4	0,8
Rien de spécial	1	0,2

**Acceptation**: la plupart des personnes interrogées (47,5%) ne faisaient pas confiances aux vaccins mis à leur disposition gratuitement par le gouvernement et n'étaient pas convaincu de leur efficacité (39,9%). Seul 16,3% était prêt à se faire vacciner ou inciter un proche à le faire. Les raisons étaient la peur d'être contaminé (41,9%) et les rumeurs sur les objectifs inavoués de la vaccination (39,8%).

**Activation**: la plupart des participants pensaient que le calendrier vaccinal doit être respecté (41,8%) et ne le respectaient que quelques fois (52,9%). En cas de retard, 30% des interrogés ramenaient l'enfant se faire vacciner malgré le retard, 24% attendaient la prochaine date et 11% arrêtaient complètement la vaccination. Pour ne pas oublier les dates de rappels, les personnes comptaient surtout sur la lecture régulière du carnet de santé (39,3%) ou le rappel par un personnel de santé (34,3%).

**Niveau d'acceptation**: à la question «Pour ou contre la vaccination », la note moyenne donnée par les participants était de 5,2 +/-2,4. Pour « Devrions-nous faire confiance au fabricant de vaccins ? », la note moyenne était de 5,0 +/- 2,4. « Faut-il se fier au ministère de la santé ? », la note moyenne était de 4,8 +/- 2,38. Pour « Je ressens l'importance d'être vacciné », moyenne 4,98 +/- 2,4 (Tableau 4).

**Tableau 4:** niveau de confiance aux vaccins proposés par le PEV des personnes interrogées à Makokou en octobre 2021

	Effectifs	%
**Faites-vous confiance aux vaccins du gouvernement ?**		
Oui	91	18,7
Non	231	47,5
Pas vraiment	164	33,7
**Pensez-vous que ces vaccins sont efficaces ?**		
Ne sait pas	2	0,4
Non pas du tout	194	39,9
Oui	112	23,0
Pas vraiment	178	36,6
**Êtes-vous prêt(e) à vous faire vacciner ou à inciter une de vos proches à se faire vacciner?**		
Ne sait pas	260	53,5
Non	147	30,2
Oui	79	16,3
**Pensez-vous qu’il est important de respecter le calendrier vaccinal ?**		
Ne sait pas	109	22,4
Pas trop	174	35,8
Oui, très important	203	41,8
**Respectez-vous scrupuleusement le calendrier vaccinal de vos enfants ou le vôtre?**		
Jamais	99	20,4
Ne sait pas	33	6,8
Oui, toujours	97	20,0
Parfois	257	52,9

**Niveau d'acceptation**: nous évaluons l'association entre le niveau d'acceptation et la provenance du chef de famille (rural ou urbain), le sexe, l'âge du chef de famille, le niveau d'instruction, la religion, le nombre d'enfants. Il y avait une association entre le milieu et le niveau d'acceptation (p <0,001), la moyenne dans le groupe rural était de 4,5 et dans le groupe urbain, de 5,6. La moyenne de l'acceptation n'était pas différente entre les deux sexes (p=0,4828). La religion était associée au niveau d'acceptation (p<0,001). Il y avait une association entre le niveau d'acceptation et le statut matrimonial (p < 0,001). Il n'y avait pas d'association entre le niveau scolaire et le niveau d'acceptation de la vaccination (p=0.345). Il y’avait une corrélation négative entre le niveau d'acceptation et le nombre d'enfants en charge (p= 0,003, le coefficient de corrélation était de -0,13). Il y’avait une association entre les connaissances sur les vaccins et les maladies, et le niveau d'acceptation (valeur p < 0,001).

**Figure 1:**
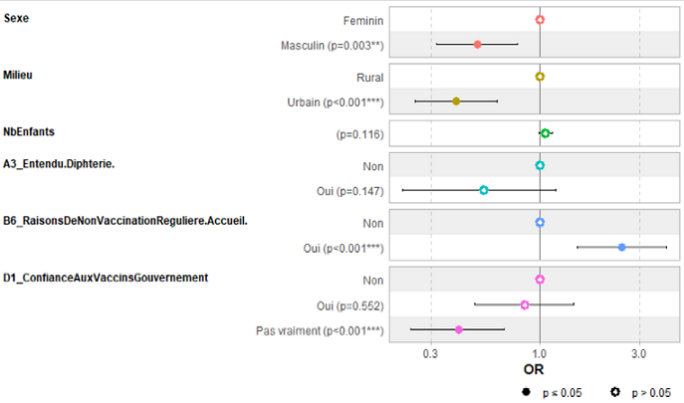
modèle final de régression logistique

**Modélisation de l'acceptation**: les variables ayant une association avec la variable niveau d'acceptation de la vaccination ou ont été introduites dans un modèle de régression logistique binaire (Tableau 5). Les caractéristiques du modèle final sont observées dans la (Figure 1). Les facteurs retenus avec une influence sur l'acceptation de la vaccination étaient le sexe, le milieu de vie (rural ou urbain), l'accueil dans les centres de vaccinations et le niveau de confiance dans les vaccins du gouvernement.

**Tableau 5:** résumé des interactions entre les différentes variables dans le modèle final

Caractéristique	OR1	95% IC1	p-valeur
**Sexe**			
Féminin	1,0	-	
Masculin	0,5	[0,3 - 0,7]	0,003
**Milieu de résidence**			
Rural	1,0	-	
Urbain	0,4	[0,2 - 0,6]	<0,001
**Nombre d’enfants**	1,0	[0,9 - 1,1]	0,12
**Avez-vous entendu parler de la diphtérie ?**			
Non	1,0	-	
Oui	0,5	[0,2 - 1,1]	0,15
**L’Accueil est la raison qui fait que votre enfant n'est pas régulièrement vacciné?**			
Non	1,00	-	
Oui	2,46	[1,51 - 4,04]	<0,001
**Faites-vous confiance aux vaccins que le gouvernement met à la disposition des populations?**			
Non	1,0	-	
Oui	0,8	[0,4 - 1,4]	0,6
Pas vraiment	0,4	[0,2 - 0,6]	<0,001


1 OR = rapport de côtes, IC = intervalle de confiance à 95%

## Discussion

Cette étude a été menée pour mesurer le niveau d'acceptation de la vaccination dans la région sanitaire Est du Gabon, où les couvertures vaccinales sont très basses afin d'identifier les facteurs qui pourraient, dans la perception de l'acte vaccinal en population générale, expliquer le faible taux de vaccination dans les ménages. Un questionnaire validé, basé sur l'approche *Five A's* a été utilisé et administré. Cette enquête n'était pas limitée aux ménages ayant des enfants, cible de la vaccination du PEV, mais à toute personne vivant dans la région où l'enquête avait été réalisée dans le but de d'avoir un profil global de la vision de la vaccination au sein de cette population. Certes cette étude a été réalisée au moment de la pandémie et la polémique sur les vaccins anti-COVID-19, les thèmes abordés dans les questions n'avaient porté que sur les vaccins et la vaccination du PEV.

Du point de vue méthodologique, nous aurions pu utiliser une autre approche, l'approche précaution adoption model [10,11]. Mais cette approche est plus complexe et nécessite une meilleure compréhension des acteurs et de l'environnement de travail. Notre questionnaire explore tout de même, les connaissances et croyances, l'accessibilité de la vaccination comme dans l'approche précaution adoption.

**Information Connaissances et source d'information:** nous avons mis en évidence que le PEV, n'était pas connu comme il le devrait dans la population, encore moins les vaccins et les maladies pour lesquels ce programme existe. Seules les maladies qui font l'objet de campagnes de vaccination, comme la rougeole, d'attention particulières par les structures sanitaires ou les personnels de santé, comme le tétanos, ou de cas d'affection grave ou de décès, comme pour la tuberculose étaient les plus connues [11-15]. Car malgré tout, les maladies les plus connues restaient celles qui étaient le plus exprimées par la population gabonaise, en termes de morbidité exprimée comme le paludisme, la diarrhée, l'hypertension artérielle, les autres, même si elles sont meurtrières, n'étaient presque pas connues [16]. Cette méconnaissance est probablement due à un manque d'information disponible et directement accessible. Par rapport aux investissements faits pour la vaccination, un effort devrait être fait pour rendre cette activité plus visible. En effet, il n'existe pas de cellule de communication au sein du PEV gabonais.

La principale source d'information sur la vaccination et les maladies évitables par la vaccination était la famille, ce qui n'est pas forcément une source fiable, alors qu'ailleurs, la principale source d'information est le personnel de santé [17]. Le personnel de santé venait en 3^e^ position, dans notre étude, après les médias traditionnels. Le PEV devrait améliorer sa stratégie de communication vers la population pour faire connaître son activité, l'intérêt de la vaccination, ses objectifs et ses buts. Il a été constaté, comme en Côte d'Ivoire, une insuffisance d'information, de communication sur la vaccination. Le personnel qui devrait réaliser cette tâche n'y est pas forcément préparé [18]. Au Gabon, la mobilisation sociale, n'est faite que lors des campagnes nationales de vaccination où ce pan est obligatoirement financé par les partenaires (UNICEF et OMS). Des plans de communication sont présentés déjà dans les Plan Pluri Annuel Complet (PPAC), il serait bon de les appliquer, car ce sont des actes de promotions de la santé pour améliorer les connaissances et la littératie en santé des populations [19-21]. Car le manque d'information sur la vaccination avait chez 26% des personnes interrogées un rôle majeur, ces programmes d'information ou d'Information Éducation et Communication (IEC) devraient être renforcés au-delà de la vaccination de routine et des autres maladies transmissibles ou non [6]. Un climat de communication permanent permettrait également de prévenir les situations où la diffusion de messages inappropriés contribuerait à semer le trouble et à l'anxiété, surtout dans le contexte de la pandémie à COVID-19 où la notion de pass vaccinal avait été introduite [22].

**Accès et accessibilité:** l'éloignement des centres de vaccination et les coûts indirects avaient été évoqués comme raisons de non vaccination dans d'autres études [23-26]. L'Ogoué-Ivivindo est l'une des plus vastes provinces du Gabon couverte à 90% de forêt, avec des voies de communications rudimentaire. Les centres de vaccinations n'y sont présents que dans les chefs-lieux de département, c'est à dire dans 4 villes dont l'accès routier est difficile, pour les populations rurales, rendant le transport coûteux et la possibilité pour les familles de faire de l'aller-retour pour vacciner leur enfant hasardeuse. En effet, faut-il dépenser, par exemple, dix mille francs CFA (soit environ 15€) par mois pendant 10 mois, coût pour un aller-retour entre le village de Mvadhy et Makokou la capitale provinciale, pour une famille qui vit en dessous du seuil de pauvreté ? Ces faits étaient observés aussi en RDC. Ainsi, à cause du déficit en route, ces enfants ne pourraient être correctement vaccinés qu'à travers des campagnes nationales régulières [27]. De plus, il serait nécessaire que les campagnes de vaccination soient précédées d'une importante mobilisation sociale pour ne pas rater les enfants dont les parents sont occupés ou au champ, afin d'éviter aux équipes de vaccinateurs des déplacement difficiles, coûteux et inutiles [28].

Pour les parents interrogés à Makokou, la vaccination des enfants se faisait le plus souvent occasionnellement, elle n'était pas planifiée, ceci pourrait être à l'origine d'occasions manquées de vaccination. Le seul élément déclencheur de rappel était la consultation du carnet ou le rappel d'un personnel de santé proche. Dans ce contexte, le respect du calendrier vaccinal serait difficile, surtout pour les parents isolés, ou avec beaucoup d'enfants à charge, comme cela avait été déjà observé ailleurs [29,30].

Au niveau organisationnel, les équipes des centres de vaccination devraient être renforcées et entraînées à accueillir de manière satisfaisante, les parents qui viennent faire vacciner leurs enfants. Le mauvais accueil des parents, le mauvais suivi des enfants ont été incriminés comme responsable de nombreux abandons et d'occasions manqués de vaccination dans d'autres études [31-34]. L'amélioration de la qualité des prestations liées à la vaccination serait donc nécessaire, comme il avait été démonté en Côte d'Ivoire et au Bénin [35-37]. Ces facteurs ont été également pointés du doigt dans le cadre de la lutte contre la rougeole en Afrique [38].

Cette enquête a été réalisée tout juste avant la campagne nationale de l'activité de vaccination intégrée (AVI) de 2021. Avant celle-ci, la dernière campagne nationale de vaccination remonte à l'année 2017 (vaccination contre la poliomyélite). Les activités de vaccination supplémentaires comme les campagnes de vaccinations ne sont pas régulières, c'est probablement la raison pour laquelle la plupart des participants n'avaient pas vacciné leurs enfants lors de la dernière campagne. Une régularité pourrait aboutir à l'amélioration de la couverture vaccinale et à la diminution de l'incidence des cas de maladies évitables par la vaccination, comme il a été observé ailleurs [39,40]. L'irrégularité ou l'arrêt d'un programme pourrait entraîner une épidémie [41,42].

En général, les personnels de santé sont accueillants et chaleureux, c'est d'ailleurs cet accueil qui déterminerait le lieu choisi pour la vaccination [18,43-45]. A ce niveau, des améliorations serait à faire comme cité plus haut. Elles pourraient prendre la forme d'un suivi personnalisé des enfants, ou d'une intégration de la vaccination dans d'autres activités de santé publiques, comme la distribution des moustiquaires ou le déparasitage systématique. La vraie difficulté serait le déficit en personnel impliqué dans la vaccination et qui du fait de circonstances comme la pandémie à COVID-19 par exemple, se retrouverait également sollicités pour la riposte [46].

**Activation:** dans notre étude, l'information et l'activation se faisaient surtout par la famille et l'entourage. Des initiatives, organisées dans certains pays, comme « la journée de la vaccination », avec une mobilisation sociale importante et l'engagement de certains leaders ou influenceurs, pourraient améliorer la perception de la vaccination dans les communautés [47].

**Acceptation:** même si le modèle final n'a pas retenu la pratique d'une religion comme facteur déterminant de l'acceptation de la vaccination, il est nécessaire que les communautés religieuses soient sensibilisées à ce sujet. Car le lien entre la couverture vaccinale et la religion pratiquées dans certains pays africains avait été mis en évidence [48]. Les leaders religieux, en tant que garant de la santé de leurs communautés respectives, devraient occuper une place importante dans la stratégie de communication faite par le PEV.

**Gestion des données:** un meilleur suivi des enfants en cours de vaccination nécessiterait également le renforcement du système d'information. Certains systèmes ont fait la preuve de leur efficacité, permettant également par ricochet d'améliorer la couverture vaccinale, comme cela s'est vu en Ouganda [49]. Il serait temps que le PEV s'engage vers une intégration des nouvelles technologies dans son système d'information.

## Conclusion

La couverture vaccinale dans une région peut s'expliquer par la manière dont est perçue la vaccination dans les ménages et les communautés. Une enquête dans la région sanitaire Est du Gabon, nous a permis de mettre en évidence le fait que le niveau d'acceptation de la vaccination était bas, tout comme la couverture des vaccins du PEV. Dans les communautés, de nombreuses connaissances étaient sur les vaccins et la vaccination erronées et nécessiteraient d'être corrigées à travers une stratégie de communication renforcée; tout comme la réorganisation du programme en lui-même et l'amélioration du profil de ses personnels. La vaccination de routine devrait continuer à s'intégrer dans la culture des populations afin de diminuer le lourd tribut payé aux maladies infectieuses par les décès des enfants dans nos pays en voie de développement.

### Etat des connaissances sur le sujet

- Les couvertures vaccinales des vaccins PEV sont globalement très basses au Gabon malgré les investissements du gouvernement;
- Certaines populations hésitent à se faire vacciner ou faire vacciner leurs enfants avec les vaccins du PEV;
- Le manque d'informations pourrait être à l'origine des couvertures vaccinales basses.

### Contribution de notre étude à la connaissance

- Évaluation du niveau d'acceptation de la vaccination dans une région du Gabon;
- Couverture vaccinale expliquée par le niveau d'acceptation de la vaccination;
- Nécessité d'améliorer les connaissances sur la vaccination de la population.

## Remerciements

Nous remercions les autorités sanitaires et administratives de l'Ogooué-Ivindo, parmi lesquels: Dr Pamphile Bekale Eloguet (*In memoriam*); Gustave Minto Mebang, Ghislain Ndohy Fouity, Bertille Balamy, Auguste Beloule; l'équipe des superviseurs et enquêteurs qui ont participé à cette étude; toutes les personnes qui ont accepté de répondre au questionnaire.
